# Self-motivated visual scanning predicts flexible navigation in a virtual environment

**DOI:** 10.3389/fnhum.2013.00892

**Published:** 2014-01-02

**Authors:** Elisabeth J. Ploran, Jacob Bevitt, Jaris Oshiro, Raja Parasuraman, James C. Thompson

**Affiliations:** Department of Psychology, George Mason UniversityFairfax, VA, USA

**Keywords:** spatial navigation, visual scanning, vicarious trial and error, attention

## Abstract

The ability to navigate flexibly (e.g., reorienting oneself based on distal landmarks to reach a learned target from a new position) may rely on visual scanning during both initial experiences with the environment and subsequent test trials. Reliance on visual scanning during navigation harkens back to the concept of vicarious trial and error, a description of the side-to-side head movements made by rats as they explore previously traversed sections of a maze in an attempt to find a reward. In the current study, we examined if visual scanning predicted the extent to which participants would navigate to a learned location in a virtual environment defined by its position relative to distal landmarks. Our results demonstrated a significant positive relationship between the amount of visual scanning and participant accuracy in identifying the trained target location from a new starting position as long as the landmarks within the environment remain consistent with the period of original learning. Our findings indicate that active visual scanning of the environment is a deliberative attentional strategy that supports the formation of spatial representations for flexible navigation.

## INTRODUCTION

Navigation through an environment to a goal location is a multifaceted task involving accessing spatial memory to identify the location of the goal, planning a route based on known properties of the environment, updating an internal representation of one’s location on the route and in the overall environment, and flexibly adjusting the route given unanticipated barriers. Navigation is made even more complex when one is learning a goal location in a new environment, and therefore must gather and integrate information on an ongoing basis for later use when returning to the same place or trying to retrace the route (Lindberg and Gärling, 1983). Given such complexities, humans employ a variety of strategies including the use of landmarks (Lipman, 1991; Chen et al., 2010), memorizing turn sequences with and without specific visual cues at decision points (Kuipers, 1978), using path integration with either egocentric (self-based) or allocentric (“bird’s eye” or environment-based) changes in heading (Gramann et al., 2005), developing an allocentric “cognitive map” (Tolman, 1948), or developing a cognitive collage which includes elements of all of the above (Tversky, 1993). Many studies indicate that some strategies, particularly those that involve orientation to the allocentric axis of the environment, lead to better navigation performance than others. However, it remains unclear what cognitive processes a navigator must use in a novel environment in order to support later navigation from novel starting positions.

Although conscious allocation of attention may be necessary to support both egocentric and allocentric knowledge (Chrastil and Warren, 2012), growing evidence suggests that attention to environmental visual features, in particular, underlies the development and use of a more flexible navigational style. Indeed, people who are better navigators are more likely to indicate relationships between buildings and objects on sketch maps of a recently learned environment (Rovine and Weisman, 1989; Billinghurst and Weghorst, 1995), remember specific intersections (Teske and Balser, 1986), and identify critical locations and landmarks that indicate turns on a learned route even if those landmarks are not as visually salient as other less critical options (Allen et al., 1979). In addition, better navigators tend to select landmarks that will be continuously useful (e.g., a playground) rather than ones that could be susceptible to changes over time (e.g., a trash bin; Kato and Takeuchi, 2003), indicating that any escalation in visual attention is combined with increases in conscious selection of the target items. Together, these findings demonstrate that successful navigation, particularly the creation and use of an environmental representation that can handle changes in starting location, relies heavily on the integration and understanding of relationships among the visual aspects of the environment. In order to reach a destination accurately, better navigators exploit the relationships between visual landmarks to identify their own location and that of their target.

Despite the abundance of evidence demonstrating differences in attention to, selection of, and memory for visual details between predefined navigation groups, there are few studies examining the causal nature of attention to visual details. Igloi et al. (2009) have shown that participants who made more rotations to scan a simple star-shaped environment with distal landmarks were also more likely to find the learned target from a novel starting point. Rotating prior to movement during search is akin to the head movements and scanning shown in rats during a variety of maze activities, a phenomenon labeled “vicarious trial and error” or VTE (Tolman, 1938). The VTE behavior originally described by Tolman was thought to reflect visual exploratory behavior as a navigator gathers information about an unfamiliar environment. According to the exploratory account, scanning behaviors should be greatest in unfamiliar environments and then decrease as the parameters of an environment become familiar. Interestingly, scanning behavior in rats becomes more likely as the difficulty of a navigation task increases and exists beyond the initial learning stage, but then drops off over time. A similar sustained level of rotations even after several experiences with the environment was found in the star maze test with human subjects (Igloi et al., 2009), suggesting that visual details in the environment may still be considered important even once familiarity has been reached.

Others have suggested that VTE-like behaviors reflect conditioned orienting and the reduction of VTE over time may be due to fatigue or other refractory processes (Spence, 1960). Alternatively, VTE may reflect the choice processes involved in evaluating the potential outcomes of two (or more) options. Eye-tracking studies have indicated that the length of fixations, and the location of the fixation in the visual field, can be predictive of behavioral choices in simple perceptual decision tasks. In addition, the duration of fixations on each option increases as the value of the two choices nears equivalency, but fixation behavior still retains its predictive nature of behavioral choice even in these difficult situations (Krajbich et al., 2010). Other studies have indicated that active, volitional exploration of novel objects improves subsequent memory performance (Voss et al., 2011). In terms of navigation, recent evidence suggests that scanning the environment reflects the investigation of alternatives based on previously learned outcomes (Johnson et al., 2012). For example, rats demonstrated more VTE behavior as the delay to receive a reward following a particular choice increased (Papale et al., 2012). When expectations about the environment are violated or altered, the navigator may simulate the potential future outcomes of different action decisions in order to predict and evaluate the possible consequences. Under this theory, visual scanning behavior should be greatest following a change in an otherwise familiar environment. Further familiarization with the environment and its possible states might then be predicted to lead to a reduction of visual scanning.

There are several important unanswered questions relating to the use of visual scanning and integration to support flexible navigation through a novel environment. First, previous studies of camera rotations indicative of visual scanning were unable to separate whether these actions were purely cognitive in nature or partially motivated and/or caused by movement planning. In the study by Igloi et al. (2009), the ability to rotate the camera and the ability to move were controlled by the same joystick and therefore mixed together in a way that makes it difficult to distinguish between rotations performed purely for visual purposes and those performed as part of a motor plan (even if that motor plan was later aborted in favor of another option). The current study separated the response commands to visually scan vs. move about the virtual environment, allowing for better quantification of conscious visual attention. Second, the results from Rovine and Weisman (1989), among others, suggest that people with better navigation skills are able to recreate relationships among visual items after experiencing novel environments only a few times. However, they fail to demonstrate a potential underlying cognitive process or behavior that may promote this phenomenon. It is possible that relationships can be found and integrated without the addition of visual scanning, but it is more likely that purposeful visual scanning will improve encoding, thereby increasing the likelihood of memory for object relationships. The current study addressed this question directly by measuring how the amount of visual scanning during navigation was related to later memory for object relationships.

Following on the basic design from Igloi et al. (2009), the current study assessed the use of visual scanning during navigation in a desktop virtual environment. Importantly, several changes were made to better separate visual scanning from movement. First, the ability to scan the environment was purposefully separated from the ability to move by requiring an extra level of motor response to allow for camera rotation. In addition, the environment was made larger and more complex to lower the likelihood that an accidental or unsure movement response would result in an approach to the target location. By separating the rotation button presses from movement responses, we could better quantify how much scanning behavior occurs without confounding it with aborted or accidental movement plans.

The second major change was the removal of rewards on *probe* trials, during which the participant must navigate to the target from a new starting location. Instead of receiving a reward upon locating the target location, participants were asked to mark the target location wherever they thought it was located. Requiring the participant to indicate the target location accomplishes three goals in assessing navigational performance. First, it eliminates the possible premature ending to a trial when a participant walks through the target intersection without knowing it. Second, it allows for quantification of how well a group of participants understands the target location by examining the accuracy of marked locations. Finally, Igloi et al. (2009) found that some participants follow a memorized sequence of moves, even when placed at a different start location than that used during training. If the end of this sequence of moves is rewarded, it may reinforce the sequential move behavior, thus limiting the likelihood that navigators will adjust their behavior later (e.g., after noticing the change in starting location). Overall, asking participants to mark the target location should create a better reflection of their knowledge of the environment and target location.

By increasing our ability to quantify visual scanning behavior, we sought to examine if visual scanning occurred primarily during the initial learning of the environment, consistent with an exploratory hypothesis of scanning behavior, or if scanning was greatest when changes to the environment occurred, consistent with the deliberative search hypothesis. Notably, these two hypotheses are not mutually exclusive. Navigators may engage in both initial exploratory and later deliberative behavior. Our goal was to attempt to quantify that behavior in way that could be integrated into a predictive model of navigational performance over time. In addition to higher accuracy locating the trained target intersection from an alternative starting location, those participants who exhibit more visual scanning should also have better memory for the relationships between the distal landmarks present around the virtual environment. This result would indicate higher levels of integration of environmental elements during self-motivated visual exploration that could then support later navigational performance.

## MATERIALS AND METHODS

### PARTICIPANTS

Ninety-four participants (57 female; mean age = 20.6 years, SD = 5.2, range = 18–53) were recruited from the George Mason University community. Data from two participants was lost due to experimenter error; two more participants failed to complete all trials. Of the remaining 90 participants, 81 (45 female) completed 75% or more of the trials before the timer ran out and were retained for further analysis. All procedures, including written informed consent, were approved by the Human Subjects Review Board at George Mason University.

### QUESTIONNAIRES

Prior to the main task, participants were asked to fill out the Santa Barbara sense of direction questionnaire (Hegarty et al., 2002) and a demographics questionnaire that included items regarding sex, age, the type of environment they experienced during their childhood, and the types of navigational experiences they had in that environment (e.g., driving, public transportation).

### NAVIGATION TASK

**FIGURE 1:**
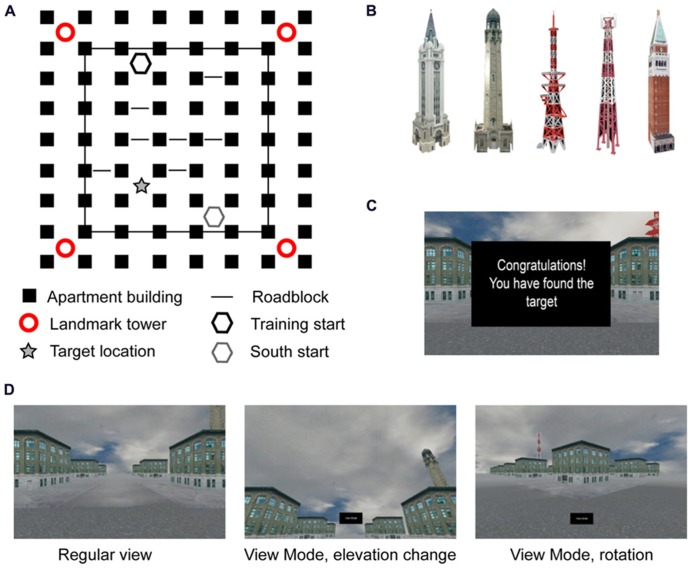
**Representation of task: (A) layout of cityscape from bird’s eye perspective; **(B)** landmark towers used in the corners, the far right tower replaced all four towers in the Uninformative Landmark condition; **(C)** feedback screen during Training trials; **(D)** examples of screen during regular navigation and “view mode” changes at an intersection**.

The main task consisted of navigation through a desktop 3-D virtual city built in-house using Blender (Blender Foundation, Amsterdam, Netherlands). The city was five intersections by six intersections in area, with a surrounding layer of intersections that the participant could see, but not actively navigate due to roadblocks obstructing the way (**Figure 1A**). All of the buildings in the city were the same nondescript apartment building, randomly rotated so that the front doors did not all face in the same direction. In addition to the apartment buildings there were four towers, one in each corner (**Figure 1B**). Two towers were radio towers, one was a traditional cell tower while the other had satellite dishes attached to the middle. The other two towers were stone buildings, one with a clock and one with a minaret. Towers were placed such that the similar towers were diagonal from each other. A fifth tower, similar in shape to the stone and clock towers, but similar in color to the radio towers, was used during Uninformative Landmark probe trials (described below). One intersection, near the satellite dish tower, was designated the target intersection for all participants. There was no physical indication in the environment that the intersection was the target; however, during Training trials participants saw a text box with the word “Congratulations” appear in front of them upon entering the target intersection from any direction (i.e., hitting the target was not dependent on entering the intersection while traveling a particular path). Importantly, the feedback box did not block the peripheral view, allowing participants to note nearby environmental features if desired (**Figure 1C**).

Before entering the test environment, participants received a brief training period on using the arrow keys for movement. Participants could move in all four directions, each with its own button; the camera turned and followed the street selected by the participant, stopping at the next intersection automatically. In addition, participants had the ability to turn on a “view mode” in any intersection prior to making a movement decision. “View mode” allowed the participant to visually explore the environment by moving the camera in 45° increments laterally and 15° increments vertically (**Figure 1D**). Participants could make as many camera movements as they would like, allowing them to turn 360° within the intersection while looking straight ahead, slightly elevated, or over the tops of the buildings. Upon turning off “view mode,” the camera immediately returned to the position in which the participant had been standing prior to looking around. Navigational movements could not be made while in “view mode.” All participants were given instruction and practice on “view mode” but were not explicitly encouraged to use it during the experiment.

The experiment began with a 180-s training phase in which the participant freely explored the test environment looking for the target intersection, at which point they would be congratulated as described above. For those participants who failed to find the target intersection within the time limit (22 out of 81, 27%), the next trial placed them one block north of the target intersection and they were instructed to press forward to reach the target, at which point they were congratulated for finding the target intersection. Throughout the environment there were roadblocks, individually set to show up on 80% of trials, preventing navigation through certain passages. The unpredictable nature of the roadblocks was designed to give participants a sense of choice in their navigational strategy, but still result in the learning over the course of the experiment a particular sequence of six moves to reach the target location as quickly as possible on most trials (the shortest route from start to the target without roadblocks was four moves, but this opportunity happened rarely).

After the initial 180-s training phase, participants were given 5 Training trials, followed by the same pre-determined interleaved sequence of 14 Training trials and 20 Probe trials (**Table 1**; similar to Igloi et al., 2009). For the purposes of this experiment, a “trial” constitutes all behaviors that occur between placement at the initial intersection until navigation into the target location (on Training trials) or marking of the target location (on Probe trials), including individual movement decisions and visual scanning of the environment. Training trials always started from the “North” start location (**Figure 1A**) and participants were instructed to find the target intersection initially identified in the training phase, at which point the feedback screen would appear on screen. The trial ended upon navigation into the target intersection or after 90 s had passed, whichever came first. Probe trials ended after participants marked an intersection or after 90 s had passed, whichever came first. Neither speed nor accuracy was emphasized; however, as stated under Participants, those participants who failed to complete 75% of the trials before the timer ran out were removed from the analysis.

**Table 1:** Trial order.

**Trial type**	1	2	3	4	5	6	7	8	9	10	11	12	13	14	15	16	17	18	19	20
	T	T	T	T	T	U	S	T	T	T	S	U	T	T	U	T	S	T	S	U
	21	22	23	24	25	26	27	28	29	30	31	32	33	34	35	36	37	38	39	
	T	S	T	S	T	U	T	S	U	S	T	U	S	T	U	T	S	U	U	

There were two types of Probe trials; for both types, participants were asked to navigate to the location of the target intersection and mark it using a button press. No feedback was given to the participant regarding his/her accuracy. Importantly, participants were told that regardless of changes in the appearance of the environment, the target location would always be in the same place as the target intersection from the Training trials (**Figure 1A**, starred location). The instructions referenced “changes in the appearance” to avoid explicitly noting that some trials would start from a new location compared to the training trials, while also avoiding surprise on the part of the participants when all four landmark towers were changed to the same uninformative tower. Therefore, participants had some knowledge that there would be changes in the environment, but were not explicitly told that probe trials existed, nor what the contents of those probe trials would be.

The first type of Probe trial was the South probe. Participants were placed in a new start location diagonally opposite of the start used in Training trials (**Figure 1A**, gray hexagon). In order to effectively navigate to the target, participants had to recognize that they were in a different starting location and they needed to adjust their route to the target accordingly. The second type of probe trial was an Uninformative Landmark trial. On Uninformative Landmark trials, all four landmark towers were replaced with the same tower (**Figure 1B**, far right) and participants were placed at the same start location as Training trials (**Figure 1A**, black hexagon). This probe was designed to test whether or not navigators retained information regarding the route to the target by removing the ability to navigate using landmark information. Roadblocks were still present in the probe trials (each set to 80% probability of appearance), but were adjusted in the South condition to match the layout of the Training trials from the new start location. Roadblocks in the Uninformative Landmark trials were in the same locations as Training trials.

### POST-TEST

Upon completing the navigation task, a surprise post-test on the map of the environment was given. Participants were handed a blank map of the environment with empty boxes in the four corners; participants were shown pictures of the four unique landmarks from the Training and South trials and were asked to place the landmarks in their correct locations at the corners of the environment. No orientation was specified for the map; participants could orient the map in any direction, only the relative relationships between the landmarks were scored (similar to the “room reconstruction” task in Skelton et al., 2000).

## RESULTS

### OVERVIEW

Navigational performance on both the South and Uninformative Landmark probe trials was measured as the distance between the location marked by participants and the actual location of the trained target intersection (“distance to target location”), measured by city blocks (similar results were obtained using Euclidean distance). Behavior on the South probe trials was also measured in terms of the distance between the location marked by participants and the location that would be reached using the six move route covertly shaped via the roadblocks during training trials (“distance to route end”); note, the trained route would lead the participant to the opposite corner of the environment from the target location on South probe trials.

To test the role of visual exploration in navigational performance, several aspects of the use of “view mode” throughout each trial (from initial intersection to marking of the target location) were considered: the camera adjustments (the total number of moves made in “view mode” both laterally and vertically), the total amount of time spent in “view mode,” and initial camera use (whether or not “view mode” was entered into immediately at the start of the trial prior to any navigational moves). These measures were highly correlated within each trial type (all *r* > 0.52, all *p* < 0.001), so only total camera adjustments were further analyzed. Final predictor variables included grand-mean centered Santa Barbara sense of direction score (SBSOD), sex, camera adjustments, trial number, and number of navigational moves. The number of navigational moves was defined as the total number of intersection-to-intersection moves made prior to marking the target (or, in rare instances, prior to the expiration of the 90-s time limit per trial).

### PERFORMANCE ON TRAINING TRIALS

**FIGURE 2:**
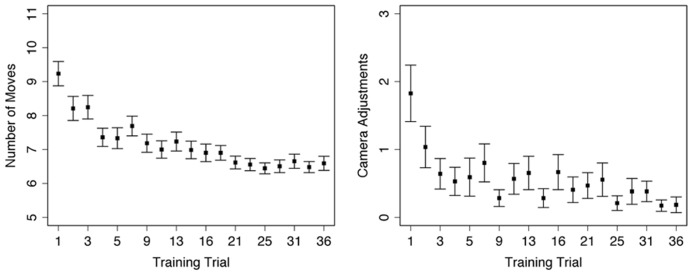
**Performance during Training trials: left – average number of moves used to reach target location within each trial; right – average number of individual changes in camera direction within each trial.** Error bars represent standard error, trial numbers on the *x*-axis match those listed in **Table 1**.

A repeated-measures one-way ANOVA on the number of moves used to reach the target location during Training trials found a significant main effect of trial number [*F*(1,18) = 12.48, *p* < 0.001], such that participants used fewer moves to reach the target location on later trials (**Figure 2**, left). Sidak-adjusted post-hoc comparisons indicated that the number of moves plateaued by the fourth trial, after which there were no longer any differences in performance with one exception. Participants made an average of 6.8 moves (SE = 0.1) to the target location starting on trial 4; the optimal route based on navigating around the roadblocks required 6 moves. The one exception was the fourth Training trial (trial #8, **Table 1**), which was the first Training trial after the introduction of the Probe trials (both Uninformative and South trials, trials #6 and 7 in **Table 1**). On this trial participants used slightly more moves (*M* = 7.7, SE = 0.3) to reach the target location, resulting in significant *post hoc* comparisons between trial 6 and the last 6 Training trials in the experiment (all *p* < 0.05). This result likely reflects that some participants noticed the differences between the Training and Probe trials, so thus explored the environment more thoroughly on the next Training trial.

A repeated-measures one-way ANOVA on total camera moves during Training trials found a significant main effect of trial number [*F*(1,18) = 4.08, *p* < 0.001]. Participants visually explored the environment less on later Training trials; however, the only significant Sidak-adjusted pairwise comparisons were between the first trial and the 15th (*p* = 0.04) and 18th (*p* = 0.03) trials. Instead, the overall decrease in camera use was a linear trend [*F*(1,18) = 11.29, *p* = 0.001] without significant decreases or increases for any given time period within the experiment (**Figure 2**, right).

Looking more closely at the first five Training trials (i.e., all Training trials prior to the introduction of the Probe trials), there was no correlation between number of moves and number of camera adjustments [*r*(79) = 0.14, *p* = 0.20], nor a correlation between number of moves and scores on the Santa Barbara sense of direction questionnaire [*r*(79) = 0.15, *p* = 0.19]. However, there was a significant correlation between SBSOD and the number of camera adjustments [*r*(79) = -0.23, *p *= 0.04); participants with better senses of direction (indicated by lower scores on the questionnaire) were more likely to use “view mode” during the initial Training trials.

### PERFORMANCE ON SOUTH TRIALS

**FIGURE 3:**
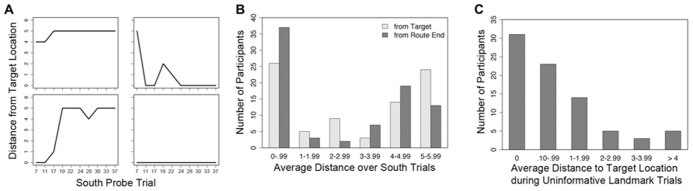
**Selected individual performance graphs and collected frequencies, trial numbers on the *x*-axis match those listed in Table 1.** **(A)** Distance from target location over 10 South probe trials for four individual subjects, the top two subjects are females and the bottom two subjects are males; **(B)** frequencies for average distance from the target location and route end among all subjects during South probe trials; **(C)** frequencies for average distance from the target location among all subjects during Uninformative Landmark trials.

On all Probe trials, participants were instructed to mark the target location from the Training trials; though not explicitly described to participants, this should have resulted in their marking the intersection near the satellite tower (**Figure 1A**, “target location”). However, if participants ignored the landmarks and instead followed the optimal sequence of six moves shaped during training, they would likely mark an intersection diagonally located across the environment; due to the reliance on the sequences of moves, we named this alternate location the “route end.” The results indicate that participants tended to go through an initial period of adjustment over the course of the first few trials, and then either consistently navigated to the correct target location or the incorrect route end location (**Figure 3B**).

There was considerable variety in navigational performance over the course of the experiment (**Figure 3A**). Instead of separating participants into groups based on a median split for sense of direction or some other measure (e.g., sex), a mixed effects model (i.e., hierarchical linear regression or growth curve modeling) was used to test whether use of “view mode” influenced accuracy on the South probe trials. This method allowed for the simultaneous inclusion of both trial-level and subject-level predictor variables to account for navigational accuracy. The influence of predictor variables on final distance from the target location was tested in two mixed models using HLM (Scientific Software International) with full maximum likelihood estimation, one model per final location. Trial-level predictor variables included number of camera adjustments and trial number (in both linear form and quadratic transformation to capture any non-linear changes in performance as the experiment progressed); subject-level predictor variables included SBSOD and sex. Due to the difference in distance from the South start location to the two potential end locations (minimum of 4 moves to the target location, 6 moves to the route end), the number of moves was not included as a predictor. First-order correlations between the predictor variables and the two performance measures can be found in **Table 2**.

**Table 2:** First-order correlations between predictor variables and performance outcomes on probe trials

	**South probe trials**		**Uninformative landmark trials**
	**Target location**	**Route end**	**Target location**
SBSOD	0.15^**^	-0.13^**^	-0.01
Camera moves	-0.37^**^	0.38^**^	0.20^**^
Trial number	-0.14^**^	0.08^*^	-0.03
Trial Number^2^	-0.11^**^	0.06	-0.02
Navigational moves	–	–	-0.02

* Denotes two-tailed Pearson’s *r*^2^ significance at *p* < 0.05; **denotes two-tailed Pearson’s *r*^2^ significance at *p* < 0.01.

Level 1 – Within individuals (i.e., across trials)

$$DistanceFromT\arg et_{ti}=\pi_{0i}+\pi_{li}\left(Trial_{ti}\right)+\pi_{2i}\left(Trial_{ti}^{2}\right)+\pi_{3i}\left(CameraAdjustments_{ti}\right)+e_{ti}$$

Level 2 – Between individuals

$$\pi_{0i}=\beta_{00}+\beta_{01}\left(Female_{i}\right)+\beta_{02}\left(SBSOD_{i}\right)+r_{0i}$$

$$\pi_{1i}=\beta_{10}+\beta_{11}\left(Female_{i}\right)+\beta_{12}\left(SBSOD_{i}\right)+r_{1i}$$

$$\pi_{2i}=\beta_{20}+\beta_{21}\left(Female_{i}\right)+\beta_{22}\left(SBSOD_{i}\right)+r_{2i}$$

$$\pi_{3i}=\beta_{30}+\beta_{31}\left(Female_{i}\right)+\beta_{32}\left(SBSOD_{i}\right)+r_{3i}$$

**FIGURE 4:**
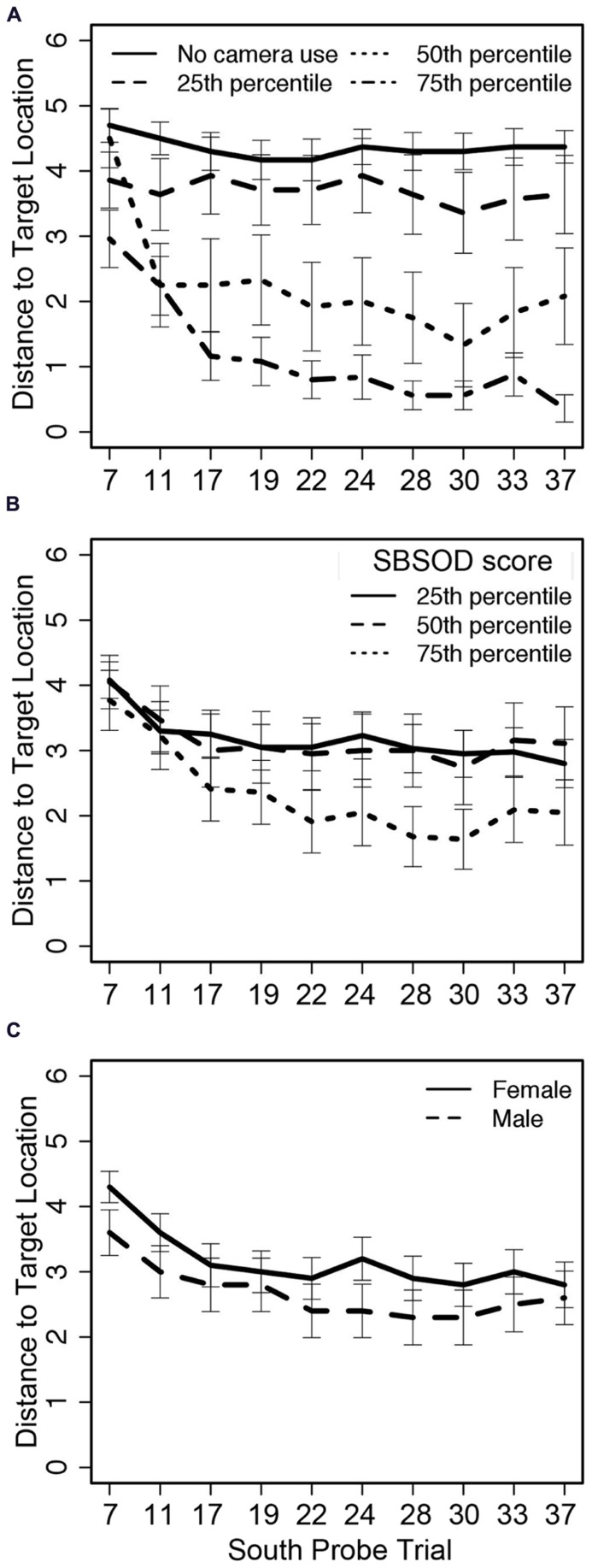
**Distance from the target location on South probe trials across 10 trials related to (A) camera adjustments, (B) sense of direction, and (C) sex; trial numbers on the *x*-axis match those listed in Table 1**.

A summary of the regression weights and associated significance is available in **Table 3**. The mixed model failed to find significant effects of SBSOD (**Figure 4B**) and sex (**Figure 4C**) on accuracy as measured by distance from the target location, but did identify significant influences of trial number (in both linear and quadratic form) and camera adjustments. Participants demonstrated learning over the course of the experiment by decreasing distance to the target location as the trials progressed, with an initial steep change in accuracy and then a plateau. Importantly, though, greater camera use predicted lower distance to the target location (i.e., better accuracy), and this effect was not moderated by sex or sense of direction (**Figure 4A**).

**Table 3:** Regression coefficients for distance to the target location on south trials.

Predictor	Coefficient	*p*-Value
**Intercept (π_0_)**
Intercept (β_00_)	4.07 (0.35)	<0.001^*^
Female (β_01_)	0.61 (0.48)	0.21
SBSOD (β_02_)	-0.01 (0.02)	0.66
**Trial (π_1_)**
Intercept (β_10_)	-0.37 (0.14)	0.01
Female (β_11_)	-0.12 (0.19)	0.55
SBSOD (β_12_)	0.01 (0.01)	0.16
**Trial^2^ (π_2_)**
Intercept (β_20_)	0.02 (0.01)	0.03
Female (β_21_)	0.01 (0.01)	0.67
SBSOD (β_22_)	-0.001 (0.001)	0.23
**Camera adjustments (π_3_)**
Intercept (β_30_)	-0.15 (0.03)	<0.001
Female (β_31_)	0.04 (0.04)	0.31
SBSOD (β_32_)	-0.001 (0.002)	0.47

* Significance for the intercept denotes that the distance from the target location on the first South trial for a male of average SBSOD who did not make any camera adjustments was significantly different from zero. Note: for all models, sex was a binary coded variable with 1 = female.

A second mixed effects model tested the predictive ability of SBSOD, sex, camera adjustments, and trial number on distance to the route end (i.e., the location participants would reach by following the optimal sequence of left/right/straight movements from the Training trial roadblock configuration while disregarding the change in starting location within the map based on relative location to the tower landmarks). The model was the same structure as the model described above for the distance to the target location, switching only the outcome variable. Again, the model failed to find significant effects of sex and sense of direction (** Table 4**); additionally, trial number (in both linear and quadratic form) no longer had a significant effect on location accuracy. Only camera adjustments were significantly predictive of final location; the results also indicated that greater camera use during the trial led the participants away from the (incorrect) route end location.

**Table 4:** Regression coefficients for distance to the route end on south trials.

Predictor	Coefficient	*p*-Value
**Intercept (π_0_)**
Intercept (β_00_)	1.46 (0.43)	0.001^*^
Female (β_01_)	-0.58 (0.60)	0.34
SBSOD (β_02_)	0.01 (0.02)	0.54
**Trial (π_1_)**
Intercept (β_10_)	0.26 (0.17)	0.14
Female (β_11_)	0.29 (0.24)	0.24
SBSOD (β_12_)	-0.01 (0.01)	0.16
**Trial^2^ (π_2_)**
Intercept (β_20_)	-0.02 (0.01)	0.18
Female (β_21_)	-0.02 (0.02)	0.31
SBSOD (β_22_)	0.001 (0.001)	0.27
**Camera adjustments (π_3_)**
Intercept (β_30_)	0.13 (0.03)	<0.001
Female (β_31_)	-0.01 (0.04)	0.84
SBSOD (β_32_)	0.001 (0.002)	0.44

* Significance for the intercept denotes that the distance from the “route end” on the first South trial for a male of average SBSOD who did not make any camera adjustments was significantly different from zero.

### PERFORMANCE ON UNINFORMATIVE LANDMARK TRIALS

A third mixed effects model assessed the influence of SBSOD, sex, camera adjustments, trial number (in both linear and quadratic form), and number of navigational moves on distance to the target location during Uninformative Landmark trials. First-order correlations between the predictor variables and the two performance measures can be found in **Table 2**. Overall, participants’ marked locations on Uninformative Landmark trials were tightly clustered around the actual target location (**Figure 3C**).

Level 1 – Within individuals (i.e., across trials)

$$Dis\tan ceFromT\arg et_{ti}=\pi_{0i}+\pi_{1i}\left(Trial_{ti}\right)+\pi_{2i}\left(Trial_{ti}^{2}\right)+\pi_{3i}\left(CameraAdjustments_{ti}\right)+\pi_{4i}\left(NavigationalMoves_{ti}\right)+e_{ti}$$

Level 2 – Between individuals

$$\pi_{0i}=\beta_{00}+\beta_{01}\left(Female_{i}\right)+\beta_{02}\left(SBSOD_{i}\right)+r_{0i}$$

$$\pi_{1i}=\beta_{10}+\beta_{11}\left(Female_{i}\right)+\beta_{12}\left(SBSOD_{i}\right)+r_{1i}$$

$$\pi_{2i}=\beta_{20}+\beta_{21}\left(Female_{i}\right)+\beta_{22}\left(SBSOD_{i}\right)+r_{2i}$$

$$\pi_{3i}=\beta_{30}+\beta_{31}\left(Female_{i}\right)+\beta_{32}\left(SBSOD_{i}\right)+r_{3i}$$

$$\pi_{4i}=\beta_{40}+\beta_{41}\left(Female_{i}\right)+\beta_{42}\left(SBSOD_{i}\right)+r_{4i}$$

The mixed model indicated no effect of trial number (in either linear or quadratic form), nor an effect of the number of navigational moves (**Table 5**). Participant sex and sense of direction also did not have an effect on performance. However, there was a significant effect of camera adjustments, such that those who looked around the environment during a trial were actually less successful at marking the target location.

**Table 5:** Regression coefficients for distance to target location on Uninformative Landmark trials.

Predictor	Coefficient	*p*-Value
**Intercept (π_0_)**
Intercept (β_00_)	0.72 (0.65)	0.27
Female (β_01_)	0.96 (0.87)	0.27
SBSOD (β02)	-0.01 (0.03)	0.65
**Trial (π_1_)**
Intercept (β_10_)	-0.07 (0.09)	0.43
Female (β_11_)	-0.02 (0.12)	0.89
SBSOD (β_12_)	-0.01 (0.004)	0.11
**Trial^2^ (π_2_)**
Intercept (β_20_)	0.003 (0.01)	0.66
Female (β_21_)	0.004 (0.01)	0.70
SBSOD (β_22_)	0.003 (0.01)	0.66
**Camera adjustments (π_3_)**
Intercept (β_30_)	0.06 (0.02)	0.01
Female (β_31_)	-0.05 (0.03)	0.10
SBSOD (β_32_)	-0.001 (0.001)	0.62
**Navigational moves (π_4_)**
Intercept (β_40_)	-0.04 (0.08)	0.44
Female (β_41_)	-0.01 (0.11)	0.92
SBSOD (β_42_)	0.01 (0.004)	0.14

### POST-TEST RESULTS

The landmark placement test was scored based on the relative configuration of the landmarks. Only 37.04% of participants placed the landmarks in the correct configuration (all four landmarks neighboring each other correctly, regardless of orientation on map); 23.46% of participants maintained the correct diagonal relationships (radio towers were placed diagonally across from each other, as were the stone towers), but one of the pairs was reversed in placement. Finally, 39.51% of participants had no discernible correct relationship among the landmarks. Each participant was given a score of 1 (no relationship), 2 (correct diagonals), or 3 (correct configuration). A linear regression was used to predict the landmark placement score based on sex, SBSOD score, total camera moves throughout all trials, and average distance to the target location during the South probe trials. Overall the model predicted a significant amount of variance in landmark placement score [*R*^2^ = 0.22, *F*(4,76) = 5.36, *p* = 0.001]. Sex [*t*(76) = -1.22, *p* = 0.23] and SBSOD [*t*(76) = 1.28, *p* = 0.20] did not have a significant impact on the outcome, nor did total camera movements [*t*(76) = 0.55, *p* = 0.58]. Only accuracy in labeling the target location on South probe trials predicted later ability to correctly place the landmarks [*t*(76) = -3.10, *p* = 0.003]. Participants who were more accurate (i.e., closer to the target location) on South probe trials were better at later placing the landmarks in the correct configuration.

## DISCUSSION

The current study examined the relationship between self-motivated visual scanning of a novel environment and both the resulting accuracy in navigation to a previously learned target and the accuracy of the mental representation of the environment after extended experience. Departing from previous studies on navigation using virtual environments, the current study required a separate set of button commands to allow for visual exploration. This aspect of the design allowed for quantification of movements made specifically for the purposes of visual scanning as opposed to accidental or abandoned movement commands. Overall, camera use predicted better accuracy when participants were placed in a new starting location as long as the landmarks in the environment available during training were still present.

The exploratory hypothesis of vicarious trial and error behavior suggests that visual scanning is used most in unfamiliar environments, as an agent is learning the parameters that define that environment, and then slowly drops off over time (Tolman, 1938). Our results demonstrate a consistent decrease in camera use during Training trials, indicating that participants became more comfortable with the environment as time went on. The deliberative search hypothesis of VTE suggests that scanning occurs when change occurs in an already familiar environment, such as when expectations about that environment are violated (Johnson et al., 2012; Papale et al., 2012). It is logical that a disruption to the navigational task may lead to extra visual scanning of the environment in order to assure the navigator that he/she has all the information necessary to perform accurately. The predictive nature of camera use on navigational accuracy from a novel starting point in our study appears to be consistent with this deliberative search hypothesis. Navigators were familiar with the target location by the first South probe trial and had decreased consistently in camera use over the first five Training trials. However, upon placement at a new start location during the South probe trials, camera use became a significant predictor of navigational accuracy. The more participants engaged in camera use, the better their resulting navigation to the target location. This suggests that successful navigation after a change in the environment relies on deliberate self-motivated visual scanning in order for the navigator to locate landmarks and cues to the target location. These results also match previous studies that have noted an increase in body rotations in a virtual environment when approaching a target from a new starting position (Igloi et al., 2009, 2010).

There were two interesting and unexpected results related to the amount of visual scanning behavior. First, camera use during Uninformative Landmark trials was predictive of worse navigational accuracy, suggesting that participants who were reliant on visual scanning during navigation were less likely to have internalized the sequence of moves that would lead them to the target location. Though the inaccuracies during Uninformative Landmark trials were small (approximately one city block difference from the actual target location), it was still unexpected to see any differences arise. This finding suggests spending attentional resources on visual scanning and encoding of certain landmarks and/or details in the environment may lead to a detriment to, or lack of an attempt of, learning sequences of moves in route form. Second, spending more time in “view mode” did not predict greater accuracy in remembering the relationships among the landmarks, as might be expected based on amount of time spent encoding the visual stimuli. Instead, participants with greater accuracy in locating the target on South probe trials had better memory for the landmark configuration. We propose that together these results suggest a cascade of cognitive action. Participants who visually scanned the environment more thoroughly were better able to locate the target intersection from a novel starting point. Completing this novel navigation successfully, in turn, allowed participants to create a better internal map of the environment, leading to better accuracy in landmark placement. Unfortunately, it is difficult to test the causal relationship of this cascade using the current experiment, as landmark placement accuracy was only assessed at the end of the experiment.

A limitation of the current experiment was the inability to identify exactly *what* the participants were seeking through visually scanning the environment. Given the non-significant effect of camera use on post-test accuracy of landmark configuration, it seems that (at least some) participants were not using their visual scanning to create an accurate memory of the overall environment. Instead, it is possible that participants could be using a beacon-like strategy (Foo et al., 2007), by focusing on only the landmark closest to the target location, without encoding the other landmarks specifically. In addition, it could be that those participants who found the navigational task to be easy were able to apply leftover attentional resources to camera use unrelated to the task; this may not have been possible for those participants who struggled with the primary task.

The next step may be to use navigation in a virtual environment in conjunction with eye-tracking or some other metric in order to separate the possible visual strategies our observed camera use may support. In addition, future research should work to identify how particular changes to the environment (e.g., local vs. distal landmarks, landmark consistency, and the magnitude of visual changes at starting points) each affect navigational behavior. Instead of focusing on categories of navigators (e.g., those with strong senses of direction vs. those without such a characteristic), it may be more important to identify what aspects of the environment promote one type of observable behavior (e.g., visual scanning) over others and the extent that the observed behavior changes navigational performance. Furthermore, this study served as the behavioral basis for a forthcoming fMRI study that may be able to identify which cognitive strategy visual scanning represents based on which brain areas are involved. We have demonstrated that visual scanning is a conscious attentional strategy that significantly relates to navigation when entering a known environment from a novel starting location. Further defining how much visual scanning affects navigation performance, during both the learning and test phases, will help identify critical periods in which navigational behaviors can be adjusted through instruction and training.

## AUTHOR CONTRIBUTIONS

Elisabeth J. Ploran, Jacob Bevitt, Jaris Oshiro, Raja Parasuraman, and James C. Thompson contributed equally to the development of the research paradigm. Jacob Bevitt and Jaris Oshiro assisted in data collection. Elisabeth J. Ploran, James C. Thompson, and Jacob Bevitt conducted the statistical analysis. Elisabeth J. Ploran and James C. Thompson wrote the report.

## Conflict of Interest Statement

The authors declare that the research was conducted in the absence of any commercial or financial relationships that could be construed as a potential conflict of interest.
